# Relationship between bone mineral density, its associated physiological factors, and tooth loss in postmenopausal Korean women

**DOI:** 10.1186/s12905-015-0218-x

**Published:** 2015-08-26

**Authors:** Chang-Suk Kim, Eun-Kyong Kim, Kyeong-Soo Lee, Hee-Kyung Lee, Youn-Hee Choi, Tae-Yoon Hwang, Jun Sung Moon

**Affiliations:** Department of Dental Hygiene, Ulsan College, Ulsan, South Korea; Department of Dental Hygiene, College of Science & Technology, Kyungpook National University, Sangju, South of Korea; Department of Preventive Medicine & Public Health, College of Medicine, Yeungnam University, Daegu, South Korea; Department of Dentistry, Yeungnam University, Daegu, South Korea; Department of Preventive Dentistry, School of Dentistry, Kyungpook National University, Daegu, South Korea; Department of Internal Medicine, Yeungnam University, Daegu, South Korea

## Abstract

**Background:**

Previous studies have proposed a relationship between bone mineral density (BMD) and oral health. However, the relationship between BMD and tooth loss in female individuals is not yet well understood. Therefore, the aim of this study was to assess the association between BMD, including its related physiological factors, and tooth loss among postmenopausal women in Korea.

**Methods:**

A total of 3,992 postmenopausal women aged 50 years or above were selected from the Fourth and Fifth Korea National Health and Nutrition Examination Surveys, which were cross-sectional in design and conducted from 2008 to 2011. The participants’ BMD and number of teeth were assessed by radiologists and dentists. Socioeconomic characteristics and female-related physiological factors, including menarche age, duration of menopause, number of pregnancies, age at first child’s birth, and duration of oral contraceptive or female hormone use, were surveyed.

**Results:**

Participants who had lower BMD had significantly fewer teeth (*p* < 0.001). Female-related physiological factors, including the duration of menopause, number of pregnancies, age at first child’s birth, duration of oral contraceptive or female hormone use, and calcium intake level, showed a significant relationship with the number of teeth. Using multiple regression analysis, BMD, duration of menopause, age at first child’s birth, and duration of female hormone use significantly influenced the number of teeth.

**Conclusions:**

BMD and its related physiological factors in female individuals showed a significant relationship with the number of teeth in postmenopausal Korean women, implicating osteoporosis as a risk factor for tooth loss in postmenopausal women.

## Background

Osteoporosis is a common metabolic bone disease, often occurring in postmenopausal women, and it is defined as a bone mineral density (BMD) at least 2.5 standard deviations (SDs) below the peak bone mass of a young, healthy, sex- and race-matched reference population, according to the World Health Organization (WHO) [1]. Bone fracture can occur in patients with osteoporosis even as a result of minor trauma to the femur, spine, or wrist [2]. It has been reported that substantial loss of BMD is caused by the decrease in estrogen secretion seen in postmenopausal women [3]. Estrogen influences the formation of osteoclast and osteoblast progenitors in bone marrow, and elicits pro-apoptotic effects on osteoclasts and anti-apoptotic effects on mature osteoblasts and osteocytes [4]. After menopause, the amount of bone resorption by osteophages is greater than the degree of bone formation by osteoblasts. Furthermore, estrogen deficiency accelerates bone turnover, thereby interfering with the absorption of calcium by the intestines and resulting in an increase in the excretion of calcium [5].

Menopause in women has also been reported to affect oral health [6]. For example, estrogen deficiency may reduce the healing ability of mucosal wounds and may contribute to a reduced salivary flow rate, which may in turn affect the periodontal tissues, including alveolar bone, in cases of poor oral hygiene [7, 8]. Severe alveolar bone loss is a well-known cause of tooth loss [9].

Many studies have reported an association between osteoporosis and BMD in the mandible [10–12]. Ward and Manson reported that osteoporosis did not cause periodontitis but increased the rate of BMD reduction [13]. Hunziker *et al.* reported that a decrease in estrogen secretion may lead to osteoporosis and BMD reduction in the mandible when periodontitis is already present [14]. In addition, Tezal *et al.* reported that skeletal BMD was associated with periodontal tissues, such as alveolar bone, and clinical attachment [15].

In light of the results of previous studies, it is necessary to assess the relationship between BMD and oral health in postmenopausal women, because an increasing number of osteoporosis patients are being seen as a result of our aging population. Cooper *et al.* suggested that the number of hip fractures occurring because of osteoporosis would rise from 1.66 million in 1990 to 6.26 million by 2050, and that approximately 50 % of hip fractures would occur in Asia [16]. According to the Fourth Korea National Health and Nutrition Examination Survey (KNHANES), 39.1 % of Korean women aged 50 years and older were diagnosed with osteoporosis in Korea [17]. In addition, the BMD of Korean women aged 20–49 years was lower when compared with that of women in the US and Japan [18]. A previous study reported on the relationship between periodontal disease, tooth loss, and BMD in a Korean population; however, the study had certain limitations, including possible bias and the inability of the study population to adequately represent the general population [19]. Therefore, we analyzed data from a nationally representative survey to overcome such limitations.

The purpose of this study was to assess the relationship between BMD and tooth loss, and to identify the determinant female-related physiological factors among postmenopausal women using data from the KNHANES.

## Methods

### Study participants

This study analyzed data from the second and third years of the Fourth KNHANES (2008–2009) and the first and second years of the Fifth KNHANES (2010–2011). Data from KNHANES was publicly available and released from Korea Center for Disease Control and Prevention. The KNHANES, a nationally representative, complex, stratified, and multistage sample survey, was conducted by the Ministry of Health and Welfare, Korea. A total of 3,992 postmenopausal women aged 50 years or older who underwent both a BMD test and oral health examination were included. The KNHANES protocol was approved by the institutional review board of the Korea Centers for Disease Control and Prevention (IRB No = 2008-04EXP-01-C, 2009-01CON-03-2C, 2010-02CON-21-C, 2011-02CON-06-C).

### Assessment of BMD

BMD was assessed by trained radiologists by evaluating the lumbar spine and left femoral neck using dual-energy X-ray absorptiometry with a fan-beam densitometer (Discovery-W, Hologic, Inc., USA). If the left femur could not be adequately measured, *e.g.*, because of surgery or fracture, then the right femur was measured. The T-score, which is a widely used measure for osteoporosis screening, was calculated using the maximal BMD data from Japanese criteria [20]. In accordance with WHO standards, a T-score above −1.0 was considered normal, between −1.0 and −2.5 was considered osteopenia, and less than −2.5 was diagnosed as osteoporosis [1].

### Assessment of tooth loss

For assessment of tooth loss, trained dentists counted the number of teeth, which was defined as the number of permanent teeth excluding the third molars at the time of the oral examination.

### Questionnaires

Each participant completed a questionnaire consisting of six items on socioeconomic characteristics (age, income, education level, body mass index (BMI), family history of osteoporosis, calcium intake, and BMD) and six items on physiological factors (menarche age, duration of menopause, number of pregnancies, age at first child’s birth, and duration of oral contraceptive and female hormone use). Income was classified into four groups according to quartiles of income level. BMI was calculated by dividing the participant’s weight by the square of their height (kg/m^2^), and this was classified into three groups (underweight: <18.5, normal: ≥18.5 and <25, and obese: ≥25). Calcium intake was divided into four groups according to quartiles of calcium intake level.

### Statistical analysis

Sampling weights were applied to compensate for sample design and non-response, and integrating weights were applied for the analysis of combined data from the Fourth and Fifth KNHANES [21]. To describe the socioeconomic and health-related characteristics of the participants, data were categorized and reported as weighted percentages. For the comparison of the difference in the number of teeth according to BMD and female-related physiological factors, the analysis was performed using one-way analysis of variance. Multiple regression analysis was performed to determine the association between BMD, its female-related physiological factors, and the number of teeth. To test interaction by age, a general linear model was used. The level of statistical significance was set at a two-tailed *p*-value of 0.05. All statistical analyses were performed using SAS version 9.1.2 (SAS Institute, Inc., Cary, NC, USA).

## Results

The distribution of socioeconomic and health-related characteristics of the participants is shown in Table 1. The mean age of the participants was 64 ± 0.2 years (range: 50–95 years). Approximately half of the participants had osteopenia (48.99 %) and osteoporosis was seen in 37.60 % of the participants. The level of subjective monthly income was almost equally distributed. The education level of the majority of participants was less than elementary school (*n* = 2,723; 65 %), and based on BMI, most participants were classified as either normal (*n* = 2,394; 61 %) or obese (*n* = 1,489; 37 %).

**Table 1:** Socioeconomic characteristics and health-related characteristics of participants

Variable	Category	N (%^a^)
Age(years)^b^	50–59	1,301(40)
	60–69	1,433(31)
	70+	1,258(29)
Subjective monthly income	Low	974(24)
	Middle-low	1,011(25)
	Middle-high	996(26)
	High	953(25)
Education level	Less than elementary school	2,723(65)
	Less than middle school	543(15)
	Less than high school	545(15)
	Graduate of college or higher	164(4.7)
Body mass index	Underweight	102(2.2)
	Normal	2,394(61)
	Obesity	1,489(37)
Family history of parents with osteoporosis	Yes	646(16)
	No	3,320(84)
Bone mineral density	Normal	490(13)
	Osteopenia	1,910(49)
	Osteoporosis	1,592(38)
Total		3,992(100)

^a^Weighted percent

^b^The mean age participants was 64 ± 0.2 years, Range: 50 ~ 95 years

The association between female-related physiological factors, BMD, and the number of teeth for all participants is shown in Table 2. Overall, participants with a longer duration of menopause, greater number of pregnancies, earlier age at first child’s birth, not taking female hormones, and with a reduced intake of calcium had significantly fewer teeth (*p* < 0.01). In the 50- to 64-year-old group, the duration of female hormone use showed an association with the number of teeth. However, for participants aged 65 years or above, the age at first child’s birth, duration of female hormone use, and calcium intake were associated with the number of teeth (*p* < 0.05).

**Table 2:** Association between female related physiological factors and the number of teeth

Variable	Category	N^a^	The number of present teeth (Mean ± SD, *p*-value^†^)					
			Total women		Women aged 50–64 years		Women aged 65 years or above	
Menarche age (years)	under 14	481	19.8 ± 0.7	0.24	24.3 ± 0.5	0.10	15.0 ± 1.3	0.29
	15–16	822	19.5 ± 0.4		24.0 ± 0.4		15.3 ± 0.6	
	17+	802	18.7 ± 0.4		23.6 ± 0.6		14.0 ± 0.6	
Menarche age^a^ age_I				0.68		0.10		0.18
Duration of menopause (years)	under 3	544	25.4 ± 0.3	**<0.01**	24.0 ± 0.6	0.07	–	0.15
	4–9	792	22.0 ± 1.0		24.2 ± 0.3		14.1 ± 2.9	
	10–14	622	22.1 ± 0.4		24.1 ± 0.5		16.8 ± 1.6	
	15+	1,874	18.1 ± 0.4		23.6 ± 1.1		15.1 ± 0.3	
Duration of menopause^a^ age_I				**<0.01**		0.35		0.90
Number of pregnancies	under 3	1,049	20.6 ± 0.5	**<0.01**	24.4 ± 0.3	0.39	16.0 ± 0.8	0.97
	4–6	1,964	19.3 ± 0.3		24.1 ± 0.2		14.5 ± 0.4	
	7+	960	18.9 ± 0.4		23.6 ± 0.6		15.3 ± 0.5	
Number of pregnancies^a^ age_I				0.59		0.63		0.40
Age at first child’s birth (years)	under 19	411	17.5 ± 0.5	**<0.01**	23.2 ± 0.7	0.43	14.1 ± 0.9	**<0.01**
	20–24	2,176	19.2 ± 0.3		23.8 ± 0.3		14.6 ± 0.4	
	25+	1,305	21.6 ± 0.3		24.4 ± 0.3		16.6 ± 0.6	
Age at first child’s birth^a^ age_I				**<0.01**		0.50		0.10
Duration of taking oral contraceptives (months)	Not taking	1,740	19.0 ± 0.3	**<0.05**	24.0 ± 0.3	0.18	14.5 ± 0.5	0.12
	under 6	319	21.0 ± 0.7		24.0 ± 0.6		17.2 ± 1.7	
	7–18	212	20.9 ± 0.7		23.3 ± 0.9		16.3 ± 1.3	
	19+	1,698	19.6 ± 0.3		24.3 ± 0.3		15.1 ± 0.4	
Duration of taking oral contraceptives^a^ age_I				**<0.01**		0.08		0.48
Duration of taking female hormone drugs(years)	Not taking	1,903	18.8 ± 0.3	**<0.01**	23.7 ± 0.3	**<0.01**	14.3 ± 0.4	**<0.01**
	under 1	304	24.1 ± 0.5		25.2 ± 0.3		20.3 ± 2.1	
	more than 1	1,771	19.7 ± 0.3		24.2 ± 0.3		15.2 ± 0.5	
Duration of taking female hormone drugs^a^ age_I				**<0.01**		0.07		0.64
Calcium intake	Q1	998	17.7 ± 0.4	**<0.01**	22.9 ± 0.5	0.11	13.2 ± 0.6	**<0.05**
	Q2	998	20.0 ± 0.4		24.4 ± 0.3		15.8 ± 0.6	
	Q3	998	20.5 ± 0.4		24.0 ± 0.3		16.4 ± 0.6	
	Q4	998	20.2 ± 0.3		24.6 ± 0.3		15.2 ± 0.6	
Calcium intake^a^ age_I				**<0.05**		0.18		0.55

^a^Exclude missing values

^†^ *p*-values obtained from oneway ANOVA using general linear model for complex survey design

age_I: to test interaction by age, participants were categorized into two age groups (50–64 years and ≥65 years

The relationship between BMD and the number of teeth is shown in Table 3. Overall, participants with a greater tendency towards osteoporosis displayed significantly fewer teeth (*p* < 0.001).

**Table 3:** Association between bone mineral density and the number of teeth

Variables	Total				Number of teeth							
					Age 50–64 years				Age ≥65 years			
	Total^a^	N	Mean ± SD	*p*-value^†^	Total^a^	N	Mean ± SD	*p*-value^†^	Total^a^	N	Mean ± SD	*p*-value^†^
Normal	3,410	436	23.4 ± 0.6	**<0.01**	1,767	375	25.2 ± 0.3	0.06	1,643	61	20.4 ± 1.7	0.06
Osteopenia		1,656	20.7 ± 0.3			1,038	23.8 ± 0.3			618	16.4 ± 0.5	
Osteoporosis		1,318	18.2 ± 0.3			354	24.0 ± 0.4			964	13.9 ± 0.4	
BMD^a^ age_I				**<0.01**				0.39				0.08

^a^Exclude missing values

^†^ *p*-values obtained from oneway ANOVA using general linear model for complex survey design

age_I: to test interaction by age, participants were categorized into two age groups (50–64 years and ≥65 years).

Multiple regression analysis was performed to determine factors affecting the number of teeth. Variables including age, high income, graduating from middle school or lower and graduating from university or higher in educational level, presence of osteoporosis, duration of menopause, age at first child’s birth, and duration of female hormone use showed significant associations with the number of teeth. As age increased by 1 year, the number of teeth decreased by 0.31. Participants who had osteoporosis had 1.54 fewer teeth when compared with healthy participants. As the duration of menopause increased by 1 year, the number of teeth decreased by 0.16. In addition, as the age at the first child’s birth and the duration of female hormone use increased by 1 year, the number of teeth increased by 0.19 and 0.03, respectively (Table 4).

**Table 4:** Multiple regression analysis of factors influencing on the number of teeth

Variable	Crude		Adjusted	
	Beta Coefficients (Standard error)	*p*-value	Beta Coefficients (Standard error)	*p*-value
Bone mineral density (ref. = normal)				
Osteopenia	−3.23(0.38)	**<0.01**	−0.70(0.42)	0.10
Osteoporosis	−8.88(0.45)	**<0.01**	−1.54(0.60)	**<0.05**
Menarche age (years)	−0.88(0.14)	**<0.01**	−0.02(0.13)	0.90
Duration of menopause (years)	−0.45(0.02)	**<0.01**	−0.16(0.05)	**<0.01**
Number of pregnancies	−0.84(0.09)	**<0.01**	0.14(0.12)	0.21
Age at first child’s birth (years)	0.69(0.06)	**<0.01**	0.19(0.07)	**<0.01**
Duration of taking oral contraceptives (months)	0.000(0.000)	0.67	0.02(0.02)	0.15
Duration of taking female hormone drugs (years)	−0.000(0.000)	0.20	0.03(0.007)	**<0.01**
Calcium intake	0.003(0.001)	**<0.01**	0.001(0.000)	**<0.01**
Age (years)	−0.57(0.02)	**<0.01**	−0.31(0.06)	**<0.01**
Subjective monthly income (ref. = low)				
Middle-low	0.91(0.50)	0.07	1.29(0.64)	**0.05**
Middle-high	0.77(0.56)	0.17	0.87(0.66)	0.18
High	2.25(0.51)	**<0.01**	2.12(0.63)	**<0.01**
Education level (ref. = less than elementary school)				
Less than middle school	6.29(0.44)	**<0.01**	1.58(0.57)	**<0.01**
Less than high school	6.82(0.46)	**<0.01**	1.42(0.68)	**<0.05**
Graduate of college or higher	8.31(0.45)	**<0.01**	2.54(0.75)	**<0.01**
			Adjusted R^2^ = 0.34	

## Discussion

In this study, we observed a significant relationship among postmenopausal women between BMD, its related determinant factors, and the number of teeth. Using multiple regression analysis we observed that female-related physiological factors, including the duration of menopause, age at first child’s birth, and duration of female hormone use, were associated with the number of teeth. To the best of our knowledge, this is the first study assessing the relationship between BMD, its related factors, and tooth loss among postmenopausal women in Korea using a national survey (KNHANES).

In our study, the association between BMD and the number of teeth was statistically significant. BMD and the number of teeth were known to be significantly affected by an increase in age. As such, we stratified and analyzed age to control it as a variable [22]. Overall, participants with a greater tendency towards osteoporosis displayed significantly fewer teeth (*p* < 0.001), which coincided with the results of a previous study by Tak *et al.* [19]. Based on this result, it is reasonable to consider that female-related physiological factors associated with osteoporosis are significantly related with the number of teeth.

Gur *et al.* reported that BMD decreased with an increase in the ‘period of menopause’ [23]. These results could be inferred from the relationship between estrogen and BMD. Notelvitz reported that osteoblasts indirectly regulate the resorption activity of osteoclasts under the influence of estrogen [24]. Simm *et al.* reported that estrogen produces growth factors and contributes to bone formation [25]. Based on these findings, hormone replacement therapy has been used in the treatment of osteoporosis [26, 27]. The results of our study showed that participants taking female hormones for more than 1 year had a greater number of teeth present than those not taking female hormones. Again, this result can be inferred from the decreased estrogen secretion occurring in the latter group. According to Hunziker *et al.*, a decrease in estrogen secretion may lead to BMD loss in the mandible in patients with periodontitis [14]. Tezal *et al.* reported that systemic bone resorption affected periodontal tissues and that a common pathway for destruction exists between systemic and periodontal bone [15]. Thus, this hypothesis supported our findings. However, unexpectedly, participants taking female hormones for more than 1 year showed a lower number of teeth present than those taking female hormones for less than 1 year. This may have resulted from the relatively small number and young age of the participants who took female hormones for less than 1 year. This can be confirmed by the fact that out of 304 participants who took female hormones for less than 1 year, 228 and 76 participants were aged 50–64 years and 65 years or above, respectively.

Nevertheless, because of the limitations of cross-sectional surveys, it may be difficult to determine a causal relationship. Therefore, prospective studies are required in the future. However, the major advantages of our study were that the participants represented the general female population of Korea, and that more reliable examination results were obtained because of the use of trained examiners, including dentists and radiologists trained by a government organization.

The results of this study showed that participants with a greater tendency towards osteoporosis had fewer teeth present. Therefore, if future prospective studies investigating the relationship between these variables are conducted, we suggest that medical doctors and dentists work together in providing information to promote overall health in postmenopausal women.

## Conclusions

The findings of this study showed that postmenopausal women with low BMD had a significantly lower number of teeth when compared with healthy women. Furthermore, BMD-related physiological factors in postmenopausal female individuals showed an association with the number of teeth, implicating osteoporosis as a risk factor for tooth loss in postmenopausal women.

## Abbreviations

BMD: Bone mineral density
BMI: Body mass index
KNHANES: Korea National Health and Nutrition Examination Survey
SD: Standard deviation
WHO: World Health Organization
